# Growth effect of tki treatment in childhood CML

**DOI:** 10.1186/1687-9856-2015-S1-P32

**Published:** 2015-04-28

**Authors:** Yeon Jin Jeon, In Ah Jung, Won-Kyoung Cho, Jae-Wook Lee, Nak-Gyun Chung, Min-Ho Jung, Bin Cho, Byung-Kyu Suh

**Affiliations:** 1 Department of Pediatrics, College of Medicine, The Catholic University of Korea, Seoul, Republic of Korea

## Aim

Childhood chronic myeloid leukemia (CML) is rare myeloproliferative disorder, and diagnosed mostly in adult, representing for 10% of all CML, and accounts for up to 2-3% of all childhood leukemia. Tyrosine kinase inhibitor (TKI), mostly Imatinib mesylate, is now used in the frontline standard treatment of CML in chronic phase. The aim of this study is to investigate the growth effect of TKI treatment in childhood CML.

## Methods

This retrospective study consisted of 20 pediatric CML patients (13 males and 7 females) received TKI treatment at Seoul and Yeouido St. Mary hospital from January 2001 to January 2014. Patients with chronic-phase CML, received TKI treatment for more than 6 months were included. Height and weight data were obtained from the patient’s medical records. The differences (Δ) of height and weight standard deviation scores (SDS) at before and after treatment were calculated and associations of factors that influence the growth were analyzed.

## Result

Seventeen patients (85.0%) had reduction in height SDS was observed. Mean age at the start of TKI was 10 years, and median follow-up was 53 months. When the mean levels of Δ height and weight SDS were analyzed, we observed significant reduction in height SDS (mean±SE, -0.35±0.33, P=0.000), but not in weight SDS (mean±SE, -0.04±0.54, P=0.723). Growth deceleration was seen predominantly in patients who started TKI at a prepubertal age compared with those who started at pubertal age or started at prepubertal age but enter puberty on treatment (mean±SE, -0.59 ±0.32 vs. -0.21±0.28, P=0.015). But no significant difference of height SDS were observed between two group depend on the TKI type. After adjusting for type of puberty, significant linear correlations with the reduction of height SDS after treatment was found for age at onset treatment (r^2^=0.381, P=0.005).

## Conclusion

After received TKI treatment in childhood CML patients, a significant number of patients experience growth deceleration. Initiation of TKI treatment in prepubertal age and age of begin treatment are associated with growth impairment. Continuous follow-up and monitoring growth after received TKI treatment in childhood CML is important for improving quality of life.

